# Human Behavior Cognition Using Smartphone Sensors

**DOI:** 10.3390/s130201402

**Published:** 2013-01-24

**Authors:** Ling Pei, Robert Guinness, Ruizhi Chen, Jingbin Liu, Heidi Kuusniemi, Yuwei Chen, Liang Chen, Jyrki Kaistinen

**Affiliations:** 1 Department of Navigation and Positioning, Finnish Geodetic Institute, FIN-02431 Masala, Finland; E-Mails: robert.guinness@fgi.fi (R.G.); ruizhi.chen@fgi.fi (R.C.); jingbin.liu@fgi.fi (J.L.); heidi.kuusniemi@fgi.fi (H.K.); yuwei.chen@fgi.fi (Y.C.); liang.chen@fgi.fi (L.C.); 2 Conrad Blucher Institute for Surveying & Science, Texas A&M University Corpus Christi, Corpus Christi, TX 78412, USA; E-Mail: ruizhi.chen@tamucc.edu; 3 Psychology of Evolving Media and Technology Research Group, Institute of Behavioural Sciences, University of Helsinki, 00014 Helsinki, Finland; E-Mail: jyrki.kaistinen@helsinki.fi

**Keywords:** sensing, location, motion recognition, LS-SVM, cognitive phone, human behavior modeling

## Abstract

This research focuses on sensing context, modeling human behavior and developing a new architecture for a cognitive phone platform. We combine the latest positioning technologies and phone sensors to capture human movements in natural environments and use the movements to study human behavior. Contexts in this research are abstracted as a Context Pyramid which includes six levels: Raw Sensor Data, Physical Parameter, Features/Patterns, Simple Contextual Descriptors, Activity-Level Descriptors, and Rich Context. To achieve implementation of the Context Pyramid on a cognitive phone, three key technologies are utilized: ubiquitous positioning, motion recognition, and human behavior modeling. Preliminary tests indicate that we have successfully achieved the Activity-Level Descriptors level with our LoMoCo (Location-Motion-Context) model. Location accuracy of the proposed solution is up to 1.9 meters in corridor environments and 3.5 meters in open spaces. Test results also indicate that the motion states are recognized with an accuracy rate up to 92.9% using a Least Square-Support Vector Machine (LS-SVM) classifier.

## 1. Introduction

Human behavior modeling and activity interpretation are of increasing interest in the information society. Social applications such as assisted living and abnormal activity detection draw a lot of attention among scientists [1]. Meanwhile, smartphone sensing technologies are nowadays developing at an incredible pace. The smartphone boasts a healthy variety of sensor options for sensing the social environment. Various locating and context related sensors and network technology are embedded into mobile phones, such as GPS, WLAN (a.k.a. Wi-Fi), cellular network antennae, Bluetooth, accelerometers, magnetometers, gyroscopes, barometers, proximity sensors, humidity sensors, temperature sensors, ambient light sensors, cameras, microphones, *etc.* With this array of input or stimulus options, coupled with capable computational and networking functions, the smartphone becomes an attractive “cognitive” platform, which has a great potential to achieve an enough high intelligence to take up on the questions of social context, such as “Where are you?”, “What are you doing?”, “How are you feeling?”, “Who are you with?”, “What is happening?”, and “Why are you here?”. This article presents an approach to sensing human behavior using a cognitive phone and summarizes the current status of our research work.

**Figure 1.**
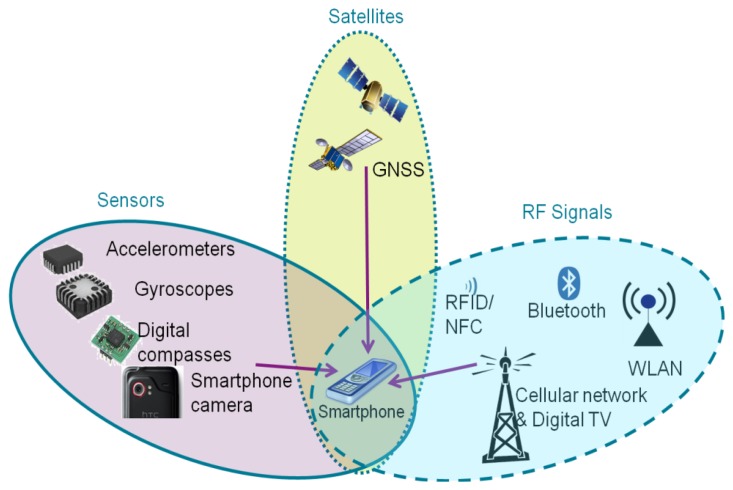
Three families of smartphone-based positioning solutions.

The question “where are you?” has been studied in the navigation and positioning fields for many decades. With the explosive growth of the capabilities in handheld computing devices, an increasing amount of research has been focused on positioning solutions using a mobile phone. In order to achieve location awareness both indoors and outdoors, as shown in the Figure 1, three families of smartphone-based positioning solutions have been studied extensively: satellite-based solutions, sensor-based solutions, and RF (radio frequency) signal-based solutions [2].

For outdoors, navigation mainly relies on satellite-based technologies. Having a wide coverage and high accuracy, standalone global navigation satellite systems (GNSS), namely for example the Global Positioning System (GPS), are the most widely applied positioning technology in smartphones. Due to the developments of visible GNSS constellations, the GNSS receiver of a smartphone has extended the positioning capability to multiple satellites systems. For instance, the Chinese phone manufacturer ZTE, together with Russian GLONASS chipset manufacturer AFK Sistema, has developed the first smart phone which embeds both GLONASS and GPS receivers. In addition, assisted GPS, also known as A-GPS or AGPS, enhances the performance of the standard GPS with additional network resources [3,4].

The existing RF infrastructures introduce some alternatives to positioning technologies on a smartphone. Positioning methods using the cellular network and WLAN are now standard features of various smartphones, such as iPhone and Android phones. Nokia has likewise developed a WiFi triangulation system, which now means that the user is more likely to get a positioning fix while indoors or in an urban canyon [5]. Furthermore, short-range RF signals such as Bluetooth [6–11] and RFID [12] are also the options for making estimates of a mobile user's location, for instance, by using proximity, fingerprinting, or triangulating.

Built-in sensors of a smartphone offer the opportunity of continuous navigation when the positioning infrastructures are unavailable. Typically, built-in sensors of a smartphone such as accelerometer, magnetometer, and gyroscope can be utilized to calculate the smartphone's speed, heading, orientation, or motion mode. The above mentioned outputs can then be applied in a pedestrian dead reckoning (PDR) algorithm to assist positioning in challenging environments where the GPS performance is poor or WLAN positioning is unavailable [13–15]. In addition, the camera in a smart phone is also a potential positioning sensor. Ruotsalainen [16,17] uses a camera on a Nokia N8 smartphone to detect the heading change of a mobile phone user. Taking advantage of the magnetometer in modern smartphones, IndoorAtlas Ltd. (Oulu, Finland) pioneers magnetic anomaly-based indoor positioning [18]. Lastly, hybrid solutions [19–21] are adopted to improve the availability and reliability of positioning by integrating all three types of solutions.

Meanwhile, human motion has been widely studied for decades, especially in recent years using computer vision technology. Poppe gives an overview of vision-based human motion analysis in [22]. Aside from vision-based solutions, sensor-based approaches are also extensively adopted in biomedical systems [23–26]. Most of the previous motion recognition related research assumed that the Micro-Electro-Mechanical Systems (MEMS) inertial sensors used are fixed on a human body in a known orientation [27–30] (e.g., in a pocket, clipped to a belt or on a lanyard) and that an error model can be obtained via training to a handful of body positions. Yang [31] uses a phone as the sensor to collect activities for off-line analysis purposes. In general, human physical activity recognition using MEMS sensors has been extensively applied for health monitoring, emergency services, athletic training, navigation, [32,33]. Since motion sensors such as accelerometers, gyroscopes and magnetometers are integrated into a smartphone, they bring the opportunity to assist navigation with knowledge about the motion of a pedestrian [34].

Together these developments suggest that locating and motion recognizing capabilities can enable the cognitive ability of sensing human behavior using a smartphone. For instance, Eagle and Pentland [35] introduce a system for sensing complex social systems using Bluetooth-enabled phones. Adams *et al.* [36] present online algorithms to extract social context: Social spheres are labeled locations of significance, represented as convex hulls extracted from GPS traces. Anderson *et al.* [37] explore the potential for use of a mobile phone as a health promotion tool. They develop a prototype application that tracks the daily exercise activities of people, using an Artificial Neural Network (ANN) to analyse GSM (Global System for Mobile communications) cell signal strength and visibility to estimate a user's movement. Choudhury and Pentland [38] develop methods to automatically and unobtrusively learn the social network structures that arise within human groups based on wearable sensors. Choudhury *et al.* [39] introduce some of the current approaches in activity recognition which use a variety of different sensors to collect data about users' activities. In this paper probabilistic models and relational information are used to transform the raw sensor data into higher-level descriptions of people's behaviors and interactions. Lane *et al.* [40] survey existing mobile phone sensing algorithms, applications, and systems. Campbell and Choudhury first introduce the Cognitive Phone concept and enumerate applications utilizing cognitive phones in [41]. Even though the term Cognitive Phone has not been officially defined yet, from the examples given by [41], the Cognitive Phone is argued to be the next step in the evolution of the mobile phone, which has the intelligence of sensing and inferring human behavior and context.

Similarly, this paper will introduce an approach to sensing human behavior, which primarily relies on ubiquitous positioning technologies and motion recognition methods. In the above cognitive research, positioning technologies such as GPS [36] and proximity [35] have been used for social context sensing. However, only outdoor activities are available because GPS is unavailable. Bluetooth proximity technology is applied for identifying users are close in terms of location. Different from the above cognition research, this approach will fully utilize seamless locating technologies on a smartphone for human behavior modeling purpose. In addition, motion states, which are usually applied for detecting personal activities [31] or some positioning purposes [33,34], will also be used for modeling human behavior in our proposed cognitive phone solution. A human behavior modeling approach named Location-Motion-Context (LoMoCo) is proposed for fusing location and motion information and inferring user's contexts. The rest of this paper is organized as follows: Section 2 provides an overview of the background of this research; Section 3 presents the proposed methods of ubiquitous positioning. We describe details of motion recognition in Section 4. Details of the LoMoCo model are represented in Section 5. Section 6 evaluates the proposed solution with experimental results. Finally, Section 7 concludes the paper and provides directions for future work.

## 2. Background and Related Work

This research is supported by a project titled INdoor Outdoor SEamless Navigation for Sensing Human Behavior (INOSENSE), funded by the Academy of Finland. The goal of the project is to carry out a study on sensing social context, modeling human behavior and developing a new mobile architecture for social applications. It aims to build a new analysis system by combining the latest navigation technologies and self-contained sensors to capture social contexts in real-time and use the system to study human movement and behavior in natural environments.

**Figure 2.**
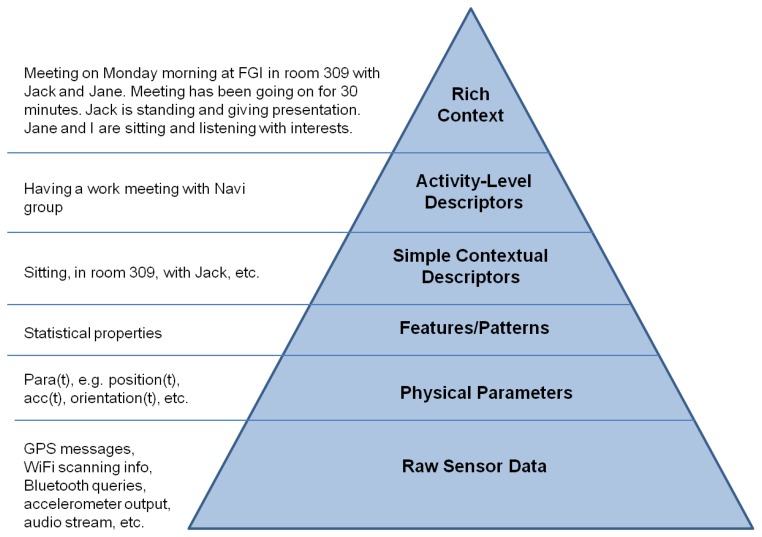
Context pyramid.

We abstract the social context as a Context Pyramid, as shown in Figure 2, where the raw data from diverse sensors is the foundation of the Context Pyramid. Based on the Raw Sensor Data, we can extract Physical Parameters such as position coordinates, acceleration, heading, angular velocity, velocity, and orientation. Features/Patterns of physical parameters are generated for further pattern recognition in the Simple Contextual Descriptors, which infer the simple context such as location, motion, and surroundings. Activity-Level Descriptors combine the simple contextual information into the activity level. On the top of the pyramid, Rich Context includes rich social and psychological contexts, which is ultimately expressed in natural language.

**Figure 3.**
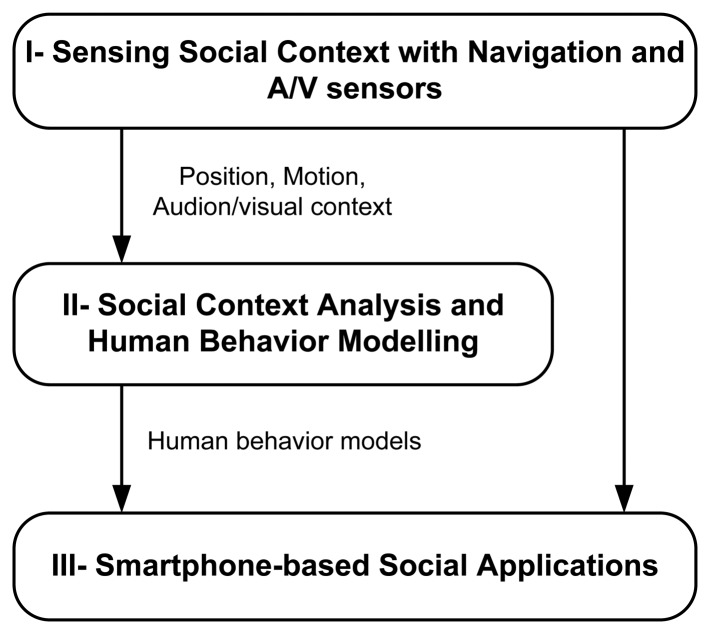
Architecture of a social application.

**Figure 4.**
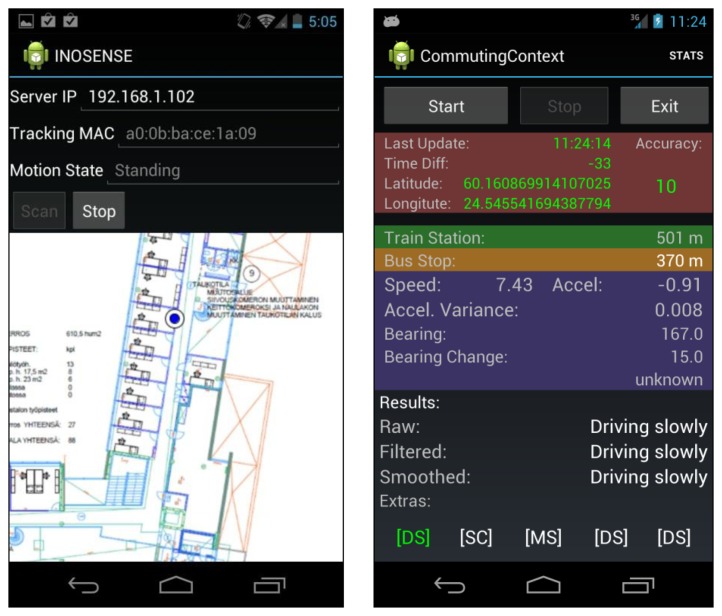
Application examples.

To implement the Context Pyramid, we break down the research into three modules as shown in Figure 3. In module I, we sense the social context with navigation and audio/visual sensors with output options such as position, motion, audio streams and visual contexts. The bottom three levels in the Context Pyramid are implemented in this module. Next, we analyze the social context and model human behavior in module II, which realizes the top three levels of the pyramid. Smartphone-based social applications ultimately use the human behavior models derived from module II, or the low level information from module I to demonstrate the use of sensing human behavior using indoor/outdoor seamless positioning technologies. Figure 4 gives two examples of mobile social applications based on the proposed architecture. On the left side is an application logging the location and motion of an employee in a workplace. It is an indoor social application using WiFi localization and motion sensors. On the right side is an application that interprets the commuting context of an employee, who works outdoors, based on location obtained from GPS and motion information from built-in sensors.

In order to implement cognitive applications, such as those shown in Figure 4, we combine the latest positioning technologies and smartphone sensors to capture human movements in natural environments and use the movement information to study human behavior. Three key technologies are applied in this research: ubiquitous positioning, motion recognition, and human behavior modeling, which will be described in the following sections.

In order to implement cognitive applications, such as those shown in Figure 4, we combine the latest positioning technologies and smartphone sensors to capture human movements in natural environments and use the movement information to study human behavior. Three key technologies are applied in this research: ubiquitous positioning, motion recognition, and human behavior modeling, which will be described in the following sections.

## 3. Ubiquitous Positioning

Location as a simple contextual descriptor in the Context Pyramid is obtained using various positioning technologies. In this research, we integrate three families of smartphone-based positioning solutions, satellite-based, sensor-based, and network-based, to achieve the location capability both indoors and outdoors. For outdoors, positioning mainly relies on satellite-based technologies. Assisted with the heading and speed estimated from smartphone sensors, the satellite-based solution can also survive in the signal-deprived environments, such as urban canyons and tunnels [42]. As outdoor positioning solutions have been fully discussed in many publications [43,44], we mainly focus on indoor environments in this paper.

### 3.1. Indoor Outdoor Detection

Different positioning technologies are applied indoors and outdoors; therefore, to fulfill the seamless positioning function, an environment-aware approach is adopted for detecting the indoor and outdoor environments. The determination of indoor/outdoor status is performed using a combination of GPS and WiFi information. The outdoor case is recognized when the number of GPS satellites and their signal-to-noise ratio is sufficiently high. Conversely, the indoor case is recognized when the GPS signals are sufficiently weak, but WiFi signal strengths are high.

As defined in Equation (1), the probability of being present indoors combines the observations of GPS and WiFi:

(1) $$P(X_{1})=\omega \cdot P_{g}\left(X_{1}|Y_{g},Z_{g}\right)+(1-\omega )\cdot P_{w}\left(X_{1}|Y_{w},Z_{w}\right)$$

where *ωϵ* [0,1] is the normalization weight of the indoor probability derived from GPS observation *P_g_* (*X_1_* | *Y_g_*, *Z_g_*), which is estimated based on the GPS signal-to-noise ratio *Y_g_* and the number of visible satellites *Z_g_*. The value of *ω* is 0.5 by default. However, it is adjustable based on prior knowledge. For instance, when a user turns off WiFi on a smartphone, *ω* can be set as 1. The indoor conditional probability *P_w_* (*X_1_* | *Y_w_*, *Z_w_*) is derived from WiFi observations including the RSSI of the strongest AP *Y_w_*, and the number of visible APs *Z_w_*. Probability lookup tables are generated for retrieving the probability based on the GPS and WiFi observations. The probability of being present outdoors can be calculated as follows:

(2) $$P(X_{2})=1-P(X_{1})$$

Considering the battery capacity limitation of a smartphone, it is a wise option to turn off unnecessary navigation sensors or decrease the sampling rate of a sensor in the procedure of seamless positioning. For instance, we suggest using a lower WiFi scanning rate in outdoor environments and suspending GPS indoors.

### 3.2. Fingerprinting Based Wireless Positioning

For indoor positioning, we adopt the fingerprinting approach of WiFi positioning. Received signal strength indicators (RSSIs) are the basic observables in this approach. The process consists of a training phase and a positioning phase. During the training phase, a radio map of probability distributions of the received signal strength is constructed for the targeted area. The targeted area is divided into a grid, and the central point of each cell in the grid is referred to as a reference point. The probability distribution of the received signal strength at each reference point is represented by a Weibull function [6,9], and the parameters of the Weibull function are estimated with the limited number of training samples.

During the positioning phase, the current location is determined using the measured RSSI observations in real-time and the constructed radio map. The Bayesian theorem and Histogram Maximum Likelihood algorithm are used for positioning [45,46].

Given the RSSI measurement vector $\vec{O}$ = {*O_1_*, *O_2_*… *O_k_*} from APs, the problem is to find the location *l* with the conditional probability *P* (*l*| $\vec{O}$) being maximized. Using the Bayesian theorem:

(3) $$\arg\max_{l}\left[P(l|\vec{O})\right]=\arg\max_{l}\left[\frac{P(\vec{O}|l)P(l)}{P(\vec{O})}\right]$$

where *P* ( $\vec{O}$ |*l*)is the probability of observing RSSI vector given a location *l*, also known as the likelihood, *P* (*l*) is the prior probability of a location *l* before observing, and *P* ( $\vec{O}$) is the marginal likelihood which indicates the probability of obtaining a given RSSI measurement vector $\vec{O}$. In this study, *P* ( $\vec{O}$) is constant for all *l*. Therefore, Equation (3) can be reduced to:

(4) $$\arg\max_{l}[P(l|\vec{O})]=\arg\max_{l}[P(\vec{O}|l)P(l)]$$

We assume that the mobile device has equal probability to be located at each reference point, thus *P* (*l*) can be considered as constant in this case. Using this assumption, Equation (4) can be simplified to:

(5) $$\arg\max_{l}[P(l|\vec{O})]=\arg\max_{l}[P(\vec{O}|l)]$$

Now it becomes a problem of finding the maximum conditional probability of:

(6) $$P(\vec{O}|l)=\prod_{n=1}^{k}P(O_{n}|l)$$

where the conditional probability *P* (*O_n_*|*l*) is derived from the RSSI distribution stored in the fingerprint database.

## 4. Motion Recognition

Motion, as another simple contextual descriptor in the Context Pyramid, can be detected by motion recognition methods. The possible motion states vary in different applications. Common motion states include sitting, standing, standing with tiny movements, fast walking, walking slowly, sharp turning, spot turning (a.k.a U-turning), gradient turning, running, using stairs, using an elevator, falling down, lying, and driving. The motion states can be further constrained in a particular use case. Given motion features, diverse classifiers can be applied for motion recognition. Feature selection and motion classification will be discussed in the following two subsections.

### 4.1. Feature Selection

This paper limits the use case to an office scenario and the applied motion states are defined as Table 1. In order to distinguish the above motion states, we currently retrieve the raw sensor data from accelerometers, gyroscope, and magnetometers built in a smartphone. The features listed in Table 2 are studied in this research. Raw data from a tri-axis accelerometer {*a_x_,a_y_,a_z_*}, gyroscope {*ω_x_,ω_y_,ω_z_*}, and magnetometer {*h_x_,h_y_,h_z_*} of a smartphone are collected, and physical parameters such as acceleration *a*, linear acceleration |*a^l^*|, horizontal acceleration *a_h_*, vertical acceleration *a_v_*, angular velocity |*ω*|, heading *h*, and so on, are calculated from the raw sensor measurements.

Thirteen features from the time domain and frequency domain are applied to the above physical parameters. The sequential forward selection (SFS) algorithm [47–49] is adopted for feature selection, and Decision Tree (DT), Linear Discriminant Analysis (LDA), and LS-SVM (Least Square-Support Vector Machines) are used as classifiers in the criterion function of SFS. The subset of features $\left\{\sigma_{a_{h}^{l}}^{2},\sigma_{a_{v}^{l}}^{2},\mu_{\omega_{x}},\mu_{\omega_{y}},\mu_{\omega_{z}},\mu_{\left|\omega \right|}\right\}$ is selected for use in a SVM classifier, which achieves the highest accuracy rate of 92.9%. The algorithm details of LS-SVM classification are described in the below subsection.

### 4.2. Classification

A supervised learning method is adopted for motion recognition. Classification algorithms such as DT, LDA, and LS-SVM are investigated in this research. After comparing these classifiers, LS-SVM is finally applied in this work because of the high accuracy of the recognition rate. Using a least squares loss function and replacing the inequality constraints with equality constraints, LS-SVM tackles linear systems instead of solving convex optimization problems in standard support vector machines (SVM), which reduces the complexity of computation [50]. In the training phase, the LS-SVM classifier constructs a hyperplane in a high-dimensional space aiming to separate the data according to the different classes. This data separation should occur in such a way that the hyperplane has the largest distance to the nearest training data points of any class. These particular training data points define the so-called margin [51,52]. These parameters can be found by solving the following optimization problem having a quadratic cost function and equality constraints:

(7) $$\arg\min J(\omega ,e)=\arg\min\left(\frac{1}{2}\omega^{T}\omega +\frac{1}{2}\gamma \sum_{i=1}^{N}e_{i}\right)$$

subject to [51]:

(8) $$y_{i}(\omega^{T}\phi (x_{i})+b)\geq 1-e_{i},i=1,\ldots ,N$$

with *e* = [*e_1_*⋯*e_N_*] *^T^* being a vector of error variables to tolerate misclassifications, sign function *y*ϵ{−1,+1}, *φ* (•): ℝ*^d^*→ℝ*^dh^* the mapping from the input space into a high-dimensional feature space of dimension *d_h_*, *ω* a vector of the same dimension as *φ* (•), *γ* is a positive regularization parameter, determining the trade-off between the margin size maximization and the training error minimization. The term *b* is the bias. In this equation, the standard SVM formulation is modified using a least squares loss function with error variables *e_i_* and replacing the inequality constraints with equality constraints [51,52]. The Lagrangian for the problem in Equations (7) and (8) is [15,52]:

(9) $$L(\omega ,b,e,\alpha )=J(\omega ,e)-\sum_{i-1}^{N}\alpha_{i}\left(y_{i}[\omega^{T}\phi (x_{i})+b]-1+e_{i}\right)$$

where *α* ϵ ℝ are the Lagrange multipliers, also support values.

Taking the conditions for optimality, we set:

(10) $$\{\begin{array}{l} \frac{\partial L}{\partial \omega }=0\to =\omega \sum_{i=1}^{N}\alpha_{i}y_{i}\varphi (x_{i}),\\ \frac{\partial L}{\partial b}=0\to \sum_{i=1}^{N}\alpha_{i}y_{i}=0,\\ \frac{\partial L}{\partial e_{i}}=0\to \alpha_{i}=ye_{i},\\ \frac{\partial L}{\partial \alpha_{i}}=0\to y_{i}[\omega^{T}\varphi (x_{i})+b]-1+e_{i}=0,i=1,\ldots ,N. \end{array}$$

Whereas the primal problem is expressed in terms of the feature map, the linear optimization problem in the dual space is expressed in terms of the kernel function [51,52]:

(11) $$\left(\begin{array}{cc} 0 & y^{T}\\ y & \Omega +\frac{1}{\gamma }I_{n} \end{array}\right)\left(\begin{array}{c} b\\ \alpha \end{array}\right)=\left(\begin{array}{c} 0\\ 1_{n} \end{array}\right)$$

where *y* = [*y_1_*⋯*y_N_*] *^T^*, *α* = [*α_1_*⋯*α_N_*] *^T^*, 1_n_ = [1⋯1] *^T^_1 × N_* and Ω ϵ ℝ*^N × N^* is a matrix with elements Ω*_ij_* = *y_i_y_j_φ* (*x_i_*)*^T^φ* (*x_j_*), with *i, j* = 1, *…, N*. Given an input vector *x*, the resulting LS-SVM classifier in the dual space is [50]:

(12) $$y(x)=\text{sign}\left(\sum_{i=1}^{N}\alpha_{i}y_{i}K(x,x_{i})+b\right)$$

where *K* (*x*,*x_i_*) = *φ* (*x*)*^T^φ* (*x_i_*) is a positive definite kernel matrix. The support values *α_i_* are proportional to the error of the corresponding training data points. This implies that usually every training data point is a support vector and no sparseness property remains in the LS-SVM formulation. Note that high support values introduce a high contribution of the data point to the decision boundary [51]. The choice of the regularization parameter and the kernel hyperparameter *δ* in case of an RBF kernel, is out of the scope for discussion in this paper. Hospodar gives an example of the kernel parameters selection in [50].

## 5. Human Behavior Modeling Based on LoMoCo Model

Modeling human behavior has great complexity, due to the wide range of activities that humans can undertake and due to the difficulties in systematically classifying these activities [15]. The approach taken in this research is to simplify the human behavior modeling using a Location-Motion-Context (LoMoCo) model which combines personal location information and motion states to infer a corresponding context based on Bayesian reasoning.

### 5.1. LoMoCo Model

**Figure 5.**
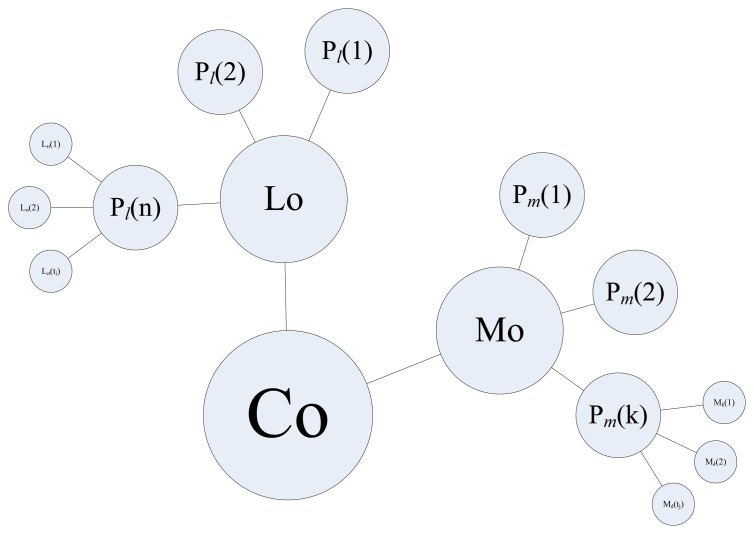
LoMoCo Model.

Given a specific context, a person always performs movements with some particular patterns. For instance, an employee usually sits in a break room while taking a break. He/she most likely stands in front of a coffee machine and shortly walks back to the office in a context of fetching coffee. In this research, we determine a context based on a LoMoCo model shown in Figure 5. In the LoMoCo model, a context (Co) is represented by location patterns (Lo) and motion patterns (Mo). Assuming that all the target contexts occur in *n* significant locations, we denote *L_n_* (*t_i_*) as a context that occurs at *L_n_* at the time epoch *t_i_*. *P_l_* (*n*) denotes the density of the context that occurs at the location *n*. A location pattern (Lo) consists of the probabilities of all the possible locations. Similarly, motion patterns (Mo) include a set of probabilities for each possible motion state. *M_k_* (*t_j_*) indicates that a context includes a motion state *M_k_* of the time epoch *t_j_*.

### 5.2. Bayes Inferring

In order to infer the context, the LoMoCo model in this paper is represented using Bayesian reasoning, which can not only determine the context but also provide with the probability of a determined class. The classifier of LoMoCo model is designed based on the Bayes rule and trained by supervised learning. In the training phase, we wish to approximate an unknown target function *P* (*Y*|*X*), where *Y* is the context predefined, and *X*={*x_1_,x_2_…x_k_*} is a vector containing observed features which are all conditionally independent of one another, given *Y*. Applying Bayes' rule, we have:

(13) $$P(Y=y_{i}|X)=\frac{P(X|Y=y_{i})P(Y=y_{i})}{\overset{N}{\underset{j=1}{\text{∑}}}P(X|Y=y_{j})P(Y=y_{j})}$$

Further, we get:

(14) $$P(Y=y_{i}|x_{1}\ldots x_{k})=\frac{\prod_{n=1}^{k}P(x_{n}|Y=y_{i})P(Y=y_{i})}{\overset{N}{\underset{j=1}{\text{∑}}}\prod_{n=1}^{k}P(x_{n}|Y=y_{i})P(Y=y_{j})}$$

where *y_i_* denotes the *i*th possible context for *Y*, and the summation in the denominator is over all legal values of the context variable *Y*. In the training phase, we use the training data to estimate *P* (*X*|*Y* = *y_i_*) and *P* (*Y* = *y_i_*) which are utilized to determine *P* (*Y* = *y_i_*|*X* = *X_z_*) for any new vector instance *X_z_*. For the classification case, we are only interested in the most probable value of *Y*, so the problem becomes:

(15) $$\arg\max y_{i}[P(Y=y_{i}|x_{i}\ldots x_{k})]=\arg\max_{y_{i}}\left[\frac{\overset{k}{\underset{n=1}{\text{∏}}}P(x_{n}|Y=y_{i})P(Y=y_{i})}{\overset{N}{\underset{j=1}{\text{∑}}}\overset{k}{\underset{n=1}{\text{∏}}}P(x_{n}|Y=y_{i})P(Y=y_{j})}\right]$$

which simplifies to the following because the denominator does not depend on a context *y_i_* :

(16) $$\arg\max y_{i}[P(Y=y_{i}|x_{i}\ldots x_{k})]={\arg\max}_{y_{i}}\left[\prod_{n=1}^{k}P(x_{n}|Y=y_{i})P(Y=y_{i})\right]$$

In the LoMoCo model, the feature vector is suggested using observations with location and motion state combined, where *X* = {*P_l_* (1), *P_l_* (2)… *P_l_* (*n*), *P_m_* (1), *P_m_* (2)… *P_m_* (*k*)}. In the case without motion or location observations, feature vector can be only location patterns where *X* = {*P_l_* (1), *P_l_* (2)… *P_l_* (*n*)} or motion patterns where *X* = {*P_m_* (1), *P_m_* (2)… *P_m_* (*k*)}. *P_l_* (*n*) and *P_m_* (*k*) are respectively calculated as:

(17) $$P_{l}(n)=\frac{\#D\{L(t_{i})=n\}}{\left|D\right|},0\leq i\leq D$$

(18) $$P_{m}(k)=\frac{\#D\{M(t_{j})=k\}}{\left|D\right|},0\leq j\leq D$$

where the # *D*{*c*}operator returns the number of samples in the set *D* that satisfy the condition *c*, and |*D*| is the total number of samples in the set *D*.

## 6. Experimental Results

**Figure 6.**
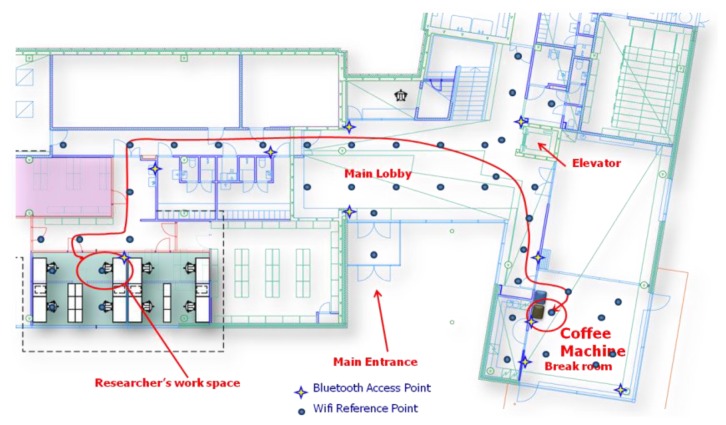
Test environment.

In order to demonstrate the proposed approach, we set up a test environment on the first floor of the Finnish Geodetic Institute (FGI), as shown in Figure 6. Positioning tests and motion recognition tests were performed in this environment to validate the positioning algorithms and motion recognition methods proposed for determining the Simple Contextual Descriptors level in Figure 2. Then, an employee-centric experiment was designed to verify whether we can achieve the Activity-Level Descriptors layer of the pyramid in Figure 2 using the LoMoCo model. Taking into account the battery capacity limitation of a smartphone, we conducted a battery drain test at last.

### 6.1. Positioning Results

This section presents the results of the above mentioned ubiquitous positioning technologies. Because outdoor positioning performance using GPS has been thoroughly discussed in many publications, for instance [43], we will mainly focus on the indoor positioning performance in this section. The test was conducted in the FGI office where forty WiFi access points are distributed among all the three floors and thirty of them might be detected on the first floor. Among all reference points, at least one and at most fourteen access points can be simultaneously observed. An Android WiFi fingerprint collection application was developed on a Samsung Galaxy Nexus. Using that application, totally 43 reference points were selected for generating the radio map for the test area. The distance between two adjective reference points is around 3–5 meters. Taking account into the factors which might affect on RSSI measurements such as the variance of RSSI observations [45], inferences from other radio systems [53], and the disturbance of human body, sixty samples were collected for each reference point from four directions during approximately 1 minute. Each direction includes about 15 samples. During the positioning tests, a tester randomly walked throughout the test zone with a built-in audio recorder in the same phone to provide a positioning reference: the tester made a mark by speaking out the name of a reference point while passing by it. In total 560 samples were collected for verifying the positioning accuracy. The entire test area is classified into three types of space: open space, corridor, and semi-open space. Open space is a large space without obstacles, such as the main lobby and break room shown in Figure 6. The corridor environment refers to a narrow hallway where a person usually is oriented in one of only two directions. Semi-open space is an open space with some obstacles, such as furniture or office partitions.

Finally, the statistical analysis results are listed in Table 3. Positioning results indicate 1.9 meters errors in corridors, 2.7 meters errors in the semi-open space, and 3.5 meters errors in the main lobby and break room. The above accuracies are high enough for room-level activity recognition.

### 6.2. Motion Recognition Results

**Figure 7.**
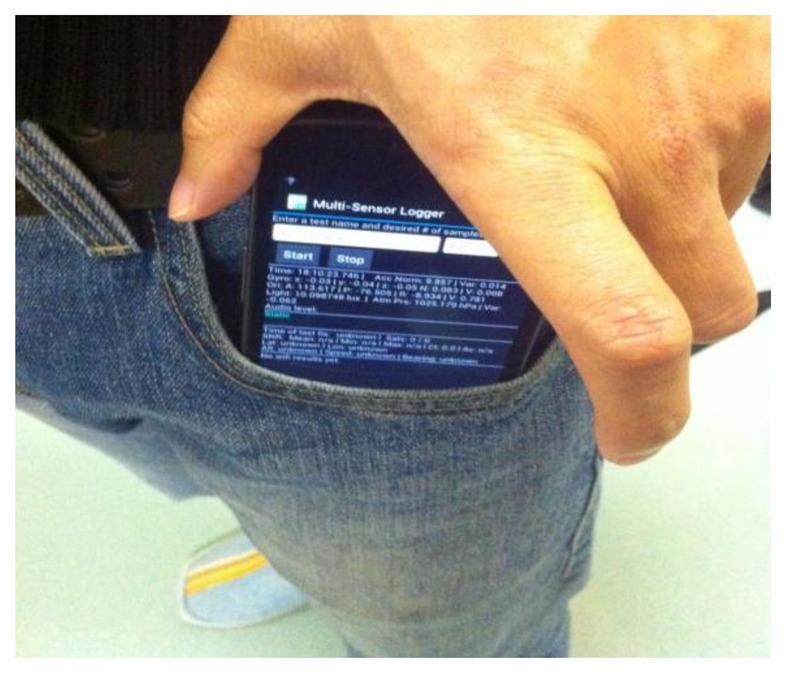
The phone in pants pocket.

The proposed motion recognition method is verified by a set of dedicated tests. Note that a phone can be placed at different positions on a user's body, which impacts the sensor data patterns. In order to reduce the complexity, the tester always kept the phone in his pants pocket and the orientation of the phone was as shown in Figure 7. Provided with a sensor data collection application (developed by the authors), four testers were involved in the sensor data collection during five days. In the FGI office building, each tester performed six motion states which are listed in Table 1. For each tester, more than 1,200 samples were collected. Thirteen types of features were extracted from the built-in accelerometers, gyroscope, and magnetometers in a smartphone.

Six motion states are detected by a Least Squares Support Vector Machines (LS-SVM) classification algorithm. The results indicate that the motion states are recognized with an accuracy rate of up to 92.9% for the test cases employed in this study. The confusion matrix in Table 4 shows that major confusions existed between sharp turning (M5) and gradient turning (M6) because these two motion states are processes depending on both the heading change and heading change rate. For example, if a user's heading changed 180 degrees in a second, the corresponding motion state will be determined as sharp turning. However, if the user changes his/her heading in more than two seconds, the motion state might be considered as gradient turning.

We also found that there are some misunderstandings between standing (M4) and sharp turning (M5). The reason is related to the training phase, where testers started a sharp turn while standing stationary, and also finished the sharp turning with a standing state. Thus, it was hard to label the sharp turning samples from the entire training data to only include the sharp turn time segment. As a result, even though a motion data set is labeled as a sharp turning state, it could include some standing states.

Despite the confusion in the turning states, the other motion states, such as sitting, normal walking, fast walking, standing, achieve a perfect success rate in the tests, and therefore can be used effectively for context determination.

### 6.3. Human Behavior Modeling Results

Activity-Level Descriptors in the Context Pyramid vary because the activity definitions and scenarios are diverse. Each different activity has its own features. As a result, it is very difficult to develop a universal model to classify activities in the Activity-Level Descriptors layer. However, location and motion are two fundamental elements of human behavior, which can be used to infer some human activities. For instance, sitting in an office might be translated as working, standing beside of a coffee machine could be considered as fetching a drink. Therefore, we proposed the LoMoCo model in Section 5. In order to demonstrate the usability of this model, we narrow down the scope of human activities to an employee's behavior with dedicated contexts in a workplace scenario as shown in Figure 6. The goal of the tests is to determine the purpose of an employee using the break room after he/she left his/her office. To simplify the problem, we define six contexts/activities in the Activity-Level Descriptors:

1. C1: fetching coffee. The tester leaves his/her office and travels through the corridors and main lobby. Then, he/she fetches coffee from a coffee machine located in the break room. Finally, he/she returns to his/her office as long as his/her coffee is ready.
2. C2: fetching water. The tester leaves his/her office and travels through the corridors, main lobby, and break room. Then, he/she fetches water from a dispenser located in the kitchen. Finally, he/she returns to his/her office as long as his/her water is ready.
3. C3: taking a break. The tester leaves his/her office and travels through the corridors and main lobby. Then he/she sits in the break room for a while. Finally, he/she returns to his/her office after a break.
4. C4: having lunch. The tester leaves his/her office and travels through the corridors, main lobby, and break room. Then, he/she prepares his/her food in the kitchen and has his/her lunch in the break room. Finally, he/she returns to his/her office after lunch.
5. C5: working. The tester sits in his/her office in most of the time. However, this context might also include some brief standing, turning, walking motion states.
6. C6: undefined context. Contexts which are not defined in the above are classified as unknown context.

**Figure 8.**
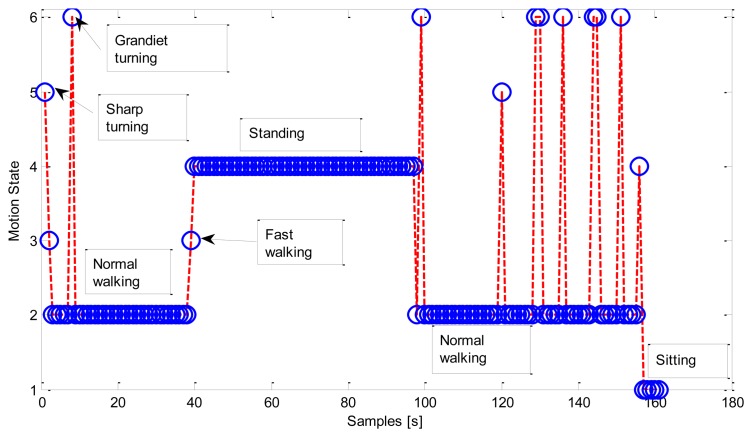
Motion states in fetching coffee context (C1).

Figure 8 gives an example of the motion states sequence occurring in a fetching coffee context. The tester firstly left the office while performing some turnings, and walked to the coffee machine. He/she stood in front of the coffee machine while fetching coffee, and walked back to his/her office after the coffee was ready. The example ended up with the tester sitting back in the office.

**Figure 9.**
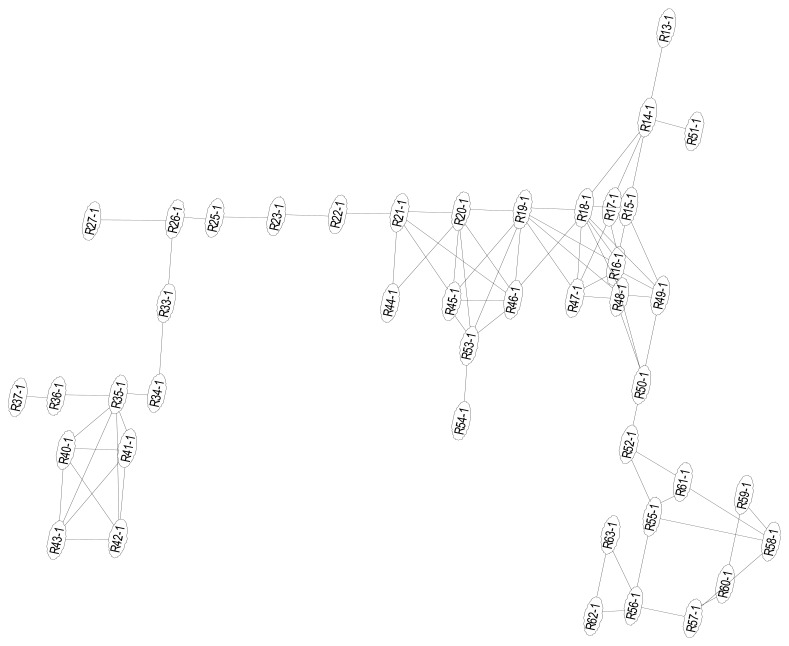
Graph of reference points.

In this test scenario, we only require room-level accuracy location. Therefore, we use the ID of a location to where the estimated reference point belongs. We organize the reference points in the test area, as shown in Figure 9, into significant locations as shown in Table 5:

Using Equation (17) in the LoMoCo model, the probability of each location is calculated for each context/activity. On the other hand, the probability of each motion state is also counted by employing Equation (18). During the training phase, twenty context samples covering four samples for each context except C6 were collected for the model training. In the testing phase, four testers performed sixty-seven contexts including fourteen C1, fifteen C2, ten C4, eight C3, and fifteen C5 context samples respectively, and five abnormal contexts which are not predefined in the LoMoCo model. The abnormal contexts included two contexts of fetching papers from a printer, two contexts of taking break in the lobby and the last one was using toilet. By applying the proposed LoMoCo model, we obtain the results as follows. Tables 6 and 7 show the results if only location features or motion features, respectively, are applied in the LoMoCo model. In the case of only location features applied, 85.5% of contexts can be correctly detected. 28.6% and 21.4% C1 contexts are mistaken as C3 and C2 respectively because those contexts have similar location patterns. Furthermore, as shown in Table 7, similar motion patterns introduce confusions between C1 and C2, C3 and C4, C3 and C5 as well. If we simultaneously take location features and motion features into account, as shown in Table 8, 90.3% of all the contexts can be correctly recognized. Abnormal contexts are classified as similar predefined contexts, for instance, the contexts of taking break in the lobby are recognized as C3, fetching a paper from a printer is labeled as fetching water or coffee. Using a toilet which is close to the office is labeled as the context of working.

### 6.4. Battery Drain Analyzing

**Figure 10.**
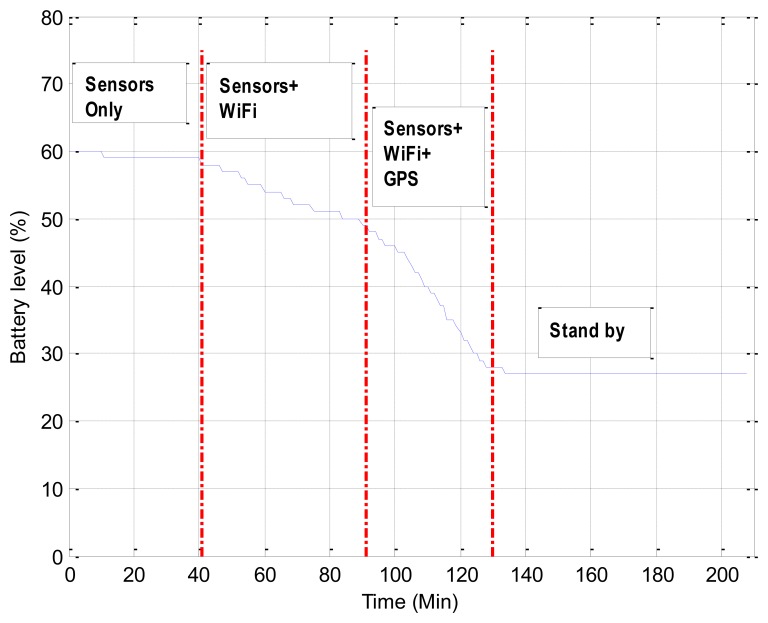
Battery drain on a smartphone.

Considering that the battery capacity is still limited, we conducted a 3.5 hours test to analyze the battery drain on a Samsung Nexus phone equipped with a 1,750 mAh Li-ion battery. A smartphone-based cognitive application as shown in the left image of Figure 4, which sampled the motion sensors around 90 Hz and scanned WiFi and GPS at about 1 Hz in the Raw Sensor Data layer of the Context Pyramid in Figure 2, was used for testing. The smartphone screen was kept off during the test. As shown in Figure 10, we started the test when 60% battery was left.

After 41 minutes with only motion sensors enabled, 2% battery was consumed. The battery was drained even faster while WiFi scanning was on. Figure 10 indicates 10% battery used in 50 minutes. The most energy-consuming case was turning on motion sensors, WiFi, and GPS insight of a smartphone simultaneously and the battery drain rate was 27.5%·h^−1^ in such circumstance. Therefore, GPS is suggested turning off or lowering the sampling rate indoors. With a fully charged battery and without any extra applications running on a smartphone, the cognitive application would constantly work 8.3 hours if only motion sensors and WiFi are turned on.

## 7. Conclusions

This research investigates context sensing, modeling human behavior, and developing a new architecture for cognitive phone platform. We combine the latest positioning technologies and sensors to capture human movements in natural environments and use the movements to study human behavior. Contexts in this research are abstracted as a Context Pyramid which includes six levels: Raw Sensor Data, Physical Parameter, Features/Patterns, Simple Contextual Descriptors, Activity-Level Descriptors, and Rich Context. To achieve understanding of the Context Pyramid on a cognitive phone, three key technologies are implemented: ubiquitous positioning, motion recognition, and human behavior modeling. Preliminary tests indicate that we have successfully achieved the Activity-Level Descriptors level with a Location-Motion-Context (LoMoCo) model with a correct rate of 90.3%. Location accuracy of the proposed solution is up to 1.9 meters errors in corridor environments and 3.5 meters errors in open space. Test results also indicate that the motion states are recognized with an accuracy rate of up to 92.9%.

Despite the fact that the motion recognition solution proposed in this paper provides a high correct motion recognition rate, the motion definition and feature selection vary from case to case. For instance, even though it is easy to confuse sharp turning with gradient turning in motion recognition, it will not effect on the classification if we merge them as one turning state in some cases. Therefore, in the future, we will investigate the motion and feature selections to find out the most effective motion states definition and features for context classification. Undefined contexts are not able to handle in the proposed LoMoCo model yet. Therefore, we will improve the model to detect abnormal behaviors. In the current stage, we successfully reach the Activity-Level Descriptors for individuals. Social activities with a group of people will be studied in the near future. Additionally, in the next step of this research work, we will focus on more complex human behavior modeling to reach the Rich Context level. The psychological state and social media context will be considered in future work.

## Figures and Tables

**Table 1.** Motion state definition.

**State**	**Definition**
M1	Sitting.
M2	Normal walking.
M3	Fast walking.
M4	Standing, this might have some tiny movements.
M5	Sharp turning (heading change: 90° < *θ* ≤ 270°).
M6	Gradient turning (heading change: −90° < *θ* ≤ 90°).

**Table 2.** Feature Definition.

**Features**	**Definition**	**Applied Physical Parameters**	**Raw Sensor Data**
*μ*	Mean	*a_x_, a_y_, a_z_, a_h_, a_v_*,|*a*|, |*a^l^*|, $a_{h}^{l}$, $a_{v}^{l}$, *ω_x_*, *ω_y_*, *ω_z_*, *ω_h_*, *ω_v_*,|*ω*|, *h_x_*, *h_y_*, *h_z_*, *h*	*a_x_*, *a_y_*, *a_z_*, *ω_x_*, *ω_y_*, *ω_z_*, *h_x_*, *h_y_*, *h_z_*
*σ*^2^	Variance	*a_x_, a_y_, a_z_, a_h_, a_v_*,|*a*|, |*a^l^*|, $a_{h}^{l}$, $a_{v}^{l}$, *ω_x_*, *ω_y_*, *ω_z_*, *ω_h_*, *ω_v_*,|*ω*|, *h_x_*, *h_y_*, *h_z_*, *h*	*a_x_*, *a_y_*, *a_z_*, *ω_x_*, *ω_y_*, *ω_z_*, *h_x_*, *h_y_*, *h_z_*
*_m_*	Median	*a_x_, a_y_, a_z_, a_h_, a_v_*,|*a*|, |*a^l^*|, $a_{h}^{l}$, $a_{v}^{l}$, *ω_x_*, *ω_y_*, *ω_z_*, *ω_h_*, *ω_v_*,|*ω*|, *h_x_*, *h_y_*, *h_z_*, *h*	*a_x_*, *a_y_*, *a_z_*, *ω_x_*, *ω_y_*, *ω_z_*, *h_x_*, *h_y_*, *h_z_*
*IQR=Q_3_-Q_1_*	Interquartile range (IQR)	*a_x_, a_y_, a_z_, a_h_, a_v_*,|*a*|, |*a^l^*|, $a_{h}^{l}$, $a_{v}^{l}$, *ω_x_*, *ω_y_*, *ω_z_*, *ω_h_*, *ω_v_*,|*ω*|, *h_x_*, *h_y_*, *h_z_*, *h*	*a_x_*, *a_y_*, *a_z_*, *ω_x_*, *ω_y_*, *ω_z_*, *h_x_*, *h_y_*, *h_z_*
$y_{\text{skewness}}=\frac{E{\left(x-\mu \right)}^{3}}{\sigma^{3}}$	Skewness	*a_x_, a_y_, a_z_, a_h_, a_v_*,|*a*|, |*a^l^*|, $a_{h}^{l}$, $a_{v}^{l}$, *ω_x_*, *ω_y_*, *ω_z_*, *ω_h_*, *ω_v_*,|*ω*|, *h_x_*, *h_y_*, *h_z_*, *h*	*a_x_*, *a_y_*, *a_z_*, *ω_x_*, *ω_y_*, *ω_z_*, *h_x_*, *h_y_*, *h_z_*
$y_{\text{kurtosis}}=\frac{E{\left(x-\mu \right)}^{4}}{\sigma^{4}}$	Kurtosis	*a_x_, a_y_, a_z_, a_h_, a_v_*,|*a*|, |*a^l^*|, $a_{h}^{l}$, $a_{v}^{l}$, *ω_x_*, *ω_y_*, *ω_z_*, *ω_h_*, *ω_v_*,|*ω*|, *h_x_*, *h_y_*, *h_z_*, *h*	*a_x_*, *a_y_*, *a_z_*, *ω_x_*, *ω_y_*, *ω_z_*, *h_x_*, *h_y_*, *h_z_*
*y_diff_* = | *y_t_*− *y_t_*_−1_|	Difference of two successive measurements	*_h_*	*h_x_*, *h_y_*, *h_z_*
*f*_1_*_st_*	1st dominant frequency	|*a*|, |*ω*|	*a_x_*, *a_y_*, *a_z_*, *ω_x_*, *ω_y_*, *ω_z_*
*f_2nd_*	2nd dominant frequency	|*a*|, |*ω*|	*a_x_*, *a_y_*, *a_z_*, *ω_x_*, *ω_y_*, *ω_z_*
*A*_*f*_1_*st*_	Amplitude of the 1st dominant frequency	|*a*|, |*ω*|	*a_x_*, *a_y_*, *a_z_*, *ω_x_*, *ω_y_*, *ω_z_*
*A*_*f*_2*nd*__	Amplitude of the 2nd dominant frequency	|*a*|, |*ω*|	*a_x_*, *a_y_*, *a_z_*, *ω_x_*, *ω_y_*, *ω_z_*
$A_{\text{scale}}=\frac{A_{f_{1st}}}{A_{f_{2nd}}}$	Amplitude scale of two dominant frequencies	|*a*|, |*ω*|	*a_x_*, *a_y_*, *a_z_*, *ω_x_*, *ω_y_*, *ω_z_*
*A_diff_* = | *A_f_*_1_*_st_* − *A_f_*_2_*_st_* |	Difference between two dominant frequencies	|*a*|, |*ω*|	*a_x_*, *a_y_*, *a_z_*, *ω_x_*, *ω_y_*, *ω_z_*

**Table 3.** Positioning results (Unit: Meter).

**Environment**	**Open Space**	**Corridors**	**Semi-open**
Mean error	3.5	1.9	2.7
RMSE	4.5	3.0	3.3
Maximum error	9.5	6.0	7.0
Minimum error	0	0	0

**Table 4.** Confusion matrix for the motion recognition from LS-SVM classifier (Unit: %).

	**M1**	**M2**	**M3**	**M4**	**M5**	**M6**
**M1**	99.5	0.5	0	0	0	0
**M2**	0	96.0	4.0	0	0	0
**M3**	0	0	100.0	0	0	0
**M4**	0	0	0	100.0	0	0
**M5**	0	0	0	16.7	64.8	18.5
**M6**	0	0	0	1.9	31.5	66.7

**Table 5.** Location definition.

**ID**	**Location**	**Reference Points ID**
**L1**	Office	R34-1∼R37-1, R40-1∼R43-1
**L2**	Corridors	R13-1∼R14-1, R22-1∼R27-1, R33-1, R49-1∼R52-1
**L3**	Main lobby	R15-1∼R21-1, R44-1∼R48-1
**L4**	Break room	R55-1∼R61-1
**L5**	Kitchen	R62-1∼R63-1

**Table 6.** Confusion matrix for the context recognition with location features (Unit: %).

	**C1**	**C2**	**C3**	**C4**	**C5**	**C6**
**C1**	50	21.4	28.6	0	0	0
**C2**	0	100	0	0	0	0
**C3**	10.0	0	90.0	0	0	0
**C4**	0	0	12.5	87.5	0	0
**C5**	0	0	0	0	100	0
**C6**	0	100	0	0	0	0

**Table 7.** Confusion matrix for the context recognition with motion features (Unit: %).

	**C1**	**C2**	**C3**	**C4**	**C5**	**C6**
**C1**	28.6	64.3	7.1	0	0	0
**C2**	0	100	0	0	0	0
**C3**	0	0	100	0	0	0
**C4**	0	0	12.5	87.5	0	0
**C5**	0	0	6.7	0	93.3	0
**C6**	0	20.0	40.0	0	40.0	0

**Table 8.** Confusion matrix for LoMoCo model (Unit: %).

	**C1**	**C2**	**C3**	**C4**	**C5**	**C6**
**C1**	64.3	35.7	0	0	0	0
**C2**	0	100	0	0	0	0
**C3**	0	0	100	0	0	0
**C4**	0	0	12.5	87.5	0	0
**C5**	0	0	0	0	100	0
**C6**	20.0	20.0	40.0	0	20.0	0
